# Exploring Resilience, Coping and Wellbeing in Women of Refugee Background Resettled in Regional Australia

**DOI:** 10.3389/fpsyg.2021.704570

**Published:** 2021-08-24

**Authors:** Clare Hawkes, Kimberley Norris, Janine Joyce, Douglas Paton

**Affiliations:** ^1^College of Health and Human Sciences, Charles Darwin University, Darwin, NT, Australia; ^2^School of Medicine (Psychology), University of Tasmania, Hobart, TAS, Australia

**Keywords:** resilience, coping, women, wellbeing, refugee

## Abstract

This study aimed to address a significant gap in the literature by investigating how Women of Refugee Background (WoRB) conceptualise resilience and identify factors that WoRB endorse as contributing to their wellbeing and coping during resettlement. Qualitative interviews were conducted with a group of 21 individuals (nine WoRB and 12 service providers). Thematic analysis identified that WoRB struggled to define resilience, with endorsed factors not fitting with current hegemonic Western understandings and theoretical understandings of resilience. The findings also highlighted how religious practice, finding a community and having a sense of meaning and contribution in their daily life were significant coping and wellbeing factors during resettlement, however, were difficult to access in regional resettlement locations. Results of the current study are discussed regarding theoretical and practical implications, taking into consideration the unique vulnerabilities experienced by WoRB resettled in regional locations of Australia.

## Introduction

Women of Refugee Background (WoRB) are continually identified as a highly vulnerable refugee population (Shishehgar et al., 2017). Throughout the refugee journey, WoRB face a high risk of experiencing gender-based violence and inequalities (Australian Government, 2020). These vulnerabilities are recognised by the United Nations High Commissioner for Refugees through the ‘Women at Risk’ visa. This visa prioritises and expedites the resettlement of WoRB and their dependents identified as being at particular risk due to their gender and lack of protection, typically provided by a male family member (Vromans et al., 2018).

Applications for the ‘Women at Risk’ visa have risen significantly over the past decade. Within an Australian context, there has been a substantial increase in the resettlement of WoRB. In the Australian 2019–2020 humanitarian program, 48.8% of visas were allocated to WoRB, with 20.4% of these being in the ‘Women at Risk’ category (Australian Government, 2020). This increase in WoRB being resettled in Australia has resulted in a call for more research to investigate the resettlement process from a gendered perspective (Sullivan et al., 2020). WoRB are frequently migrating into host countries whose social, cultural and institutional characteristics are very different from their country of origin (Darychuk and Jackson, 2015). This means that WoRB face a multitude of issues affecting their quality of life. This includes a history of persecution and trauma in their country of origin, a migration process that disproportionally exposes them to gender-based violence and inequalities, and arrival in countries generally ill-prepared to manage the consequent and complex mental issues and facilitate adaption in an alien culture. Research investigating factors contributing to the mental health and wellbeing of WoRB from a gendered perspective during resettlement are urgently needed to inform policy and practice in ways that can culminate in the development of meaningful, proactive approaches to facilitating adaptation and wellbeing in WoRB (Sullivan et al., 2020).

The majority of research focusing on WoRB mental health needs has been conducted through a psychopathology lens, focusing on diagnosable mental health conditions, symptomology, stressors and their determinates which increase the likelihood of WoRB experiencing mental distress during resettlement (Sullivan et al., 2020). This research has provided a greater understanding of the sequelae associated with mental distress experienced by WoRB resettled in Australia, including pre-migration traumatic experiences, the chronic instability and uncertainty during migration, as well as the multitude of post-migration resettlement stressors, which can adversely impact WoRB mental health (Sullivan et al., 2020). However, it has resulted in limited research focusing on factors associated with positive adaptation, coping and wellbeing in WoRB during resettlement (Schweitzer et al., 2007; Sherwood and Liebling-Kalifani, 2012; Sullivan et al., 2020).

Gaining a deeper understanding of wellbeing and positive adaptation in WoRB has been deemed an important area of future enquiry as it not only holds the potential to depathologise the mental health narratives of WoRB during resettlement (Lenette et al., 2013; Sullivan et al., 2020) but also offers insights into the factors that facilitate positive and sustainable resettlement experiences. One concept which is gaining more attention within research focusing on refugee populations, which aligns more closely with the concept of positive mental health, is the construct of resilience (Li et al., 2013; Beiser, 2014; Siriwardhana et al., 2014).

Resilience is a term that is associated with ongoing debate within psychological literature surrounding its definition and generalisability to cross-cultural populations (Denckla et al., 2020). While there is no consensus surrounding how to define, or measure, resilience, it is becoming more widely acknowledged that resilience is a complex and dynamic process, which spans multiple levels of functioning and is associated with the interaction between multiple systems, across the individual and community level (Denckla et al., 2020). Gaining a deeper understanding of the complex process which is resilience has been identified as being imperative to assist with developing a greater knowledge around protective processes, particularly for vulnerable populations (Sleijpen et al., 2017), including WoRB.

A recent review (Hawkes et al., 2020) identified and synthesised research focusing on resilience in WoRB. The factors which have been endorsed by WoRB as contributing to their resilience included as: religion and connection to culture, as well as their children, family connection and social support. WoRB also indicated that their culture of origin was a key factor in explaining why their children and family were so pivotal in their resilience, in which strong links to family and the sense of pride they derived from raising their children stemmed from their culture of origin and traditional values (Hawkes et al., 2020). In addition to identifying factors endorsed by WoRB as contributing to their resilience, the review aimed to identify the theoretical frameworks used within research investigating resilience in WoRB. The review identified 14 different theoretical frameworks; however, none of the applied theoretical frameworks adequately accounted for the majority of factors endorsed by WoRB. Overall, the review highlighted that greater consideration is needed when utilising a resilience framework developed for Western-based populations in research involving refugee populations. This is because the application of hegemonic Western-centric conceptualisations of resilience (as a construct) and associated theoretical frameworks increases the likelihood that research findings will not reflect the realities of WoRB (Lenette, 2011). Within this, the application of some theoretical frameworks associated with resilience does not acknowledge the diverse socio-cultural (e.g., collectivistic vs. individualistic cultural orientations) and socio-political (e.g., ethnic repression and persecution) contextual factors, derived from heritage country and migration factors and experiences, which influence people’s wellbeing and resilience outcomes (Ni et al., 2014). Therefore, research attempting to understand resilience and wellbeing in WoRB needs to take a more culturally grounded approach, with WoRB having the opportunity to define their own concepts of what constitutes resilience and wellbeing, and identify factors that contribute to it (Hawkes et al., 2020). Overall, it is imperative that research not only investigates ‘what’ factors are associated with greater coping and resilience, but ‘how’ these factors help refugee women adapt (Shishehgar et al., 2017), through methodologies that prioritise the experience of WoRB in a way which enhances their capacity to meaningfully contribute and shape academic discourse (Pearce et al., 2017).

Therefore, the current study aimed to explore the concept of, and factors contributing to, resilience, wellbeing and coping in WoRB resettled in a regional location of Australia. In doing so, this work holds the potential to inform future policy and practices that can more realistically seek to increase the likelihood of positive adaption and enhanced and sustained wellbeing in WoRB during resettlement and beyond. The two main questions explored in the current study are as: (1) how do WoRB define and understand resilience? and (2) what factors contribute to the wellbeing and coping of WoRB resettled in regional Australia?

## Materials and Methods

### Ethical Consideration

Research in Western resettlement countries involving refugee populations poses several ethical challenges (Clark-Kazak, 2017). Informed consent is a challenge in research involving refugee populations due to individuals of refugee backgrounds often having limited English fluency and comprehension. Further, refugee populations may often have varying levels of literacy in their first language, which can impact obtaining written informed consent (Block et al., 2013; Clark-Kazak, 2017). Due to limited English fluency and comprehension, research involving individuals from a refugee background may use interpreters. Using interpreters come with its only limitations, including the researcher’s ability to uphold confidentiality, as other individuals (i.e., interpreters) have been involved in the data collection process (Clark-Kazak, 2017), the current study addresses any concerns surrounding confidentiality and using interpreters by conducting the interview in English.

Ethical challenges in the current study were also minimised by the selection criterion: being able to speak English at a level where the interview could be conducted in English and providing the participants the opportunity to give either written consent, oral consent or both (with seven out of the nine WoRB opting to provide oral consent to participate in the study). The application of these selection criteria aimed to increase the likelihood that the WoRB participating understood the purposes of the study, the associated risks and benefits, and thus allowing them to provide informed consent. Ethics approval for the current study was obtained through the Tasmanian Social Sciences Human Research Ethics Network (H0017941; H20021) and Human Research Ethics Committee at Charles Darwin University (H19003; H19087).

### Design

The current study used a qualitative methodological framework consisting of individual semi-structured interviews guided by a set of open-ended questions (Table 1). All participants were provided the opportunity to review the questions before starting the interview.

**Table 1:** Initial open-ended guiding questions.

What does the term ‘resilience’ mean to you?^*^
How do you see/understand resilience in WoRB in Tasmania?
What factors do you believe contribute to resilience in WoRB?
What factors help them cope during resettlement?

* =asked to only WoRB.

As discussed below in the results section, ‘resilience’ was a term that many WoRB participating in the current study struggled to define or understand. Due to this, time was spent exploring similar terms which could be used as a substitute before the interview starting. All participants identified the terms ‘coping’ and ‘wellbeing’ as terms that they understood. As such, these terms were substituted for the term ‘resilience’ for WoRB who identified that they did not know what the term ‘resilience’ meant.

### Sampling and Recruitment

The current study used purposive sampling techniques in the initial stages, with subsequent participants identified *via* snowball sampling techniques. Participants were consciously selected based on their capacity to contribute to the goals of the research and included both individuals who identified as WoRB, as well as volunteers and service providers who provide support to WoRB. Service providers and volunteers were invited to participate as they play a key role in supporting WoRB during the initial stages of resettlement and influence factors associated with psychological wellbeing (Sabouni, 2019). Service providers and volunteers could identify as male or female to participate.

All participants needed to be over the age of 18 and speak a level of conversational English to participate in the research. The location and time of the interview were determined before the interview *via* telephone. Interviews were conducted in services, participant homes and private study rooms at public libraries. WoRB who agreed to participate in the study were offered a $20 gift voucher as compensation for their time. All interviews were conducted between May 2019 and August 2020.

Data collection was paused between February 2020 and June 2020 due to the COVID-19 pandemic. Data collection was re-commenced in July 2020 following ethics approval. At the time of recommencement, Tasmania had not had a community-acquired case of COVID-19 for 40days, and borders were closed to other states and territories in Australia, with hotel quarantine being mandatory for entering the state.

### Participants

A total of 21 individuals participated in the interviews (nine WoRB and 12 service providers; four individuals in volunteer-based roles and eight in paid roles). Further, participant demographic information was not collected, and hence not reported. This was to ensure the confidentiality and anonymity of participants and meet ethical requirements. This is due to the research being conducted in a regional location, and the participants having unique characteristics (i.e., being a WoRB or working/volunteering for one of the limited refugee support services), which increases the likelihood that they would be more identifiable to local stakeholders, than members of the general population.

All interviews were conducted by the first author (a female clinical psychologist), face to face, ranging from 45–75min in length and were audio-recorded. Each participant was interviewed once, and individually, resulting in a total of 21 interviews being conducted. No participants were known to the researcher before meeting, and time was spent developing rapport prior to commencing the interview. Audio recordings from interviews were transcribed verbatim and interviewees were provided the opportunity to review the transcript for comment and/or correction.

### Data Analysis

Data saturation, the point where no new interview themes emerged, determined the final number of interviews required for the study. Data were continuously analysed throughout the data collection period using NVivo 12 software (Edhlund and McDougall, 2019). An audit trail was kept throughout the research process to aid the researcher in identifying when data saturation was reached. Within the current study, by interview 17, the audit trail entries illustrated that the list of new themes began to decline, until there were no new themes identified from the 21^st^ interview; hence, this was deemed the last interview.

As the current research focused on reporting the experiences and reality of participants, transcripts were analysed utilising Braun and Clarke’s (2006) six-step thematic analysis framework, utilising inductive thematic description at the semantic level which was further underpinned by an essential/realist approach. NVivo qualitative data analysis software (version 12; Edhlund and McDougall, 2019) was utilised for data management. Reporting was guided by the Consolidated Criteria for Reporting Qualitative Research (Tong et al., 2007).

Within the preliminary research plan, it was anticipated that interviews from WoRB and service providers would need to be analysed and reported separately. However, throughout the iterative analysis process, it became evident that interviews from both WoRB and service providers fitted under the same emerging themes. Therefore, all interviews were analysed and reported together, as has been done in similar research involving service providers and refugee populations (Savic et al., 2013, 2016; Smith et al., 2020).

## Results

Two major overarching themes were identified within the data; proactive strengths-based approaches to dealing with adversity and factors contributing to wellbeing and coping during resettlement.

### Proactive Strengths-Based Approaches to Dealing With Adversity

Several WoRB expressed that although they heard the term ‘resilience’ used often, it was a concept that they did not have a clear understanding of

‘Resilience – that’s a term I hear used all the time but I do not know what it means’ – WoRB 3

Other WoRB identified that for them, ‘resilience’ was an entity that helped them ‘cope’ and continue to move forward

‘Yes I have heard about it, and I think it is something that makes us deal with and cope with’ – WoRB 7

‘Well, resilience, to me, is to have the strength to keep looking forward, if I were to, like, sum it up’ – WoRB 1

‘It’s almost just like that onefoot in front of the other, and looking forward’ – WoRB 8

This ability and strength to keep moving forward were highlighted as being within the day-to-day tasks, as WoRB faced daily struggles and challenges during resettlement

‘Their capacity to continue to overcome the challenges that they face in their every day – it’s in the practical’ – Service Provider 1

‘They [WoRB] are incredibly resilient because to face this every single day. I do not know many people who actually could face that level of adversity’ – Service Provider 10

Resilience, and the capacity to continue moving forward, within the resettlement context was expressed as stemming from their past experiences

‘I’ve encountered things that no basic 20-year-old should have to. You know, like, I made the decision to seek asylum for my own safety with my partner at the age of, maybe, 23, but that was one of the hardest things to do. And then while we were waiting for asylum, which was 1year, we did not know anything about whether, you know, we were going to be able to stay, or that we were going to be safe – That’s resilience’ – WoRB 4

‘We all come here looking for a better life, and that’s where… I guess, for me, that’s where that resilience comes from, you know, knowing that, you know, I’ve come here, I’ve made it, I’m safe, and that this is my opportunity to make a better life for myself’ – WoRB 6

‘I feel like experience contributes a lot. I know that’s where I get a lot of my strength from, knowing that, you know, I’ve been in that situation, and it’s so much different now’ – WoRB 1

Overall, resilience was a difficult concept to define for many WoRB who participated in the current study to define. For the WoRB who had heard of resilience, it was a concept which encapsulated moving forward and coping. This was highlighted as not being isolated to the ‘big events’ but rather was something that WoRB pulled upon every day as they faced adversity in everyday basic tasks during resettlement. WoRB also highlighted how their past experiences play a large role in their present-day ‘resilience’, and how their past experiences of adversity play a pivotal role in moving forward.

### Factors Contributing to Wellbeing and Coping During Resettlement

The WoRB interviewees expressed several factors which contributed to their wellbeing and coping and resilience during resettlement, falling under three sub-themes: engaging in religious practice, finding a community and sense of meaning and contribution.

#### Engaging in Religious Practice

WoRB emphasised the importance of their religious practice during resettlement, and how engaging in religious practice provided them with meaning, strength and community

‘Religion, especially religion. Religion is very important in our family’ – WoRB 5

‘When I am feeling down, I pray’ – WoRB 4

‘Our people often get together in someone’s house, or maybe book a hall, so they are able to get together to celebrate important festivals and religious event and that is also how they are able to get more connected with each other’ – WoRB 7

Service providers supporting WoRB during resettlement also emphasised the importance of engaging in religious practices during resettlement, which may not be initially conceptualised as an important coping or resilience factor from a western perspective

‘Spirituality, they are fiercely attached to their religious beliefs and I suspect that underpins their resilience’ – Service Provider 12

‘They turn to the few things that are their strengths, so faith – unbelievably strong and that’s something in our western world that’s … I’m sure it must be challenging for them because we are all a bit – ‘hah, religion!’. It must be really hard because it is a key for survival. So turning to God, whatever that god might be, turning to their scriptures, playing to their strengths’ – Service Provider 1

Despite religion being identified as a key factor in coping and wellbeing during resettlement for WoRB, interviewees highlighted that often there was no placed of worship attached to their faith in the regional location which they were resettled

‘There is no mosque here, so we have to go to a community centre, so we have time to pray’ – WoRB 5

‘Saturday, Sunday, no church… it’s full stress’ – WoRB 2

‘We cannot go and practice our religion because there is no mosque’ – WoRB 6

The lack of places of worship for some religious denominations in regional resettlement locations was expressed as a gross oversight in government policy, suggesting that there is limited consideration surrounding what factors will assist WoRB cope and achieve wellbeing during regional resettlement

‘[Practicing Religion] is what’s going to actually give us the wellbeing we need, and give us the opportunity and our children to be able to keep that practise ongoing, which will help us settle – WoRB 1

‘It’s that acknowledgement and opportunity to regularly practise their cultural experiences or traditions. As a counter to that, in Launceston, for example, we have a small number of Muslim communities. There is no prayer centre in Launceston so they come here and they are allocated to Launceston and there is nowhere they can go to pray and there are no faith leaders’ – Service Provider 1

#### Finding a Community

The WoRB and service providers emphasised that finding a community as imperative for WoRB is ongoing wellbeing during resettlement.

‘To have a community, sense of community, anyway, because you leave that behind’ – WoRB 1

‘Everyone here is my community… it does not matter where they come from. They are my community…I am happy here in my country… I decide yes, I stay here’ – WoRB 2

‘Building linkages with either their own ethnic group, or with a wider community. So, some sort of collective support’ – Service Provider 12

‘I think it’s important they find a community: whether it’s, you know, their ethnic community, or a new Australian community, like a neighbourhood group, or an interest group. I just think they need to find others who can provide them with support and encouragement’- Service Provider 5

For many WoRB, this sense of community stemmed from a connection with a community from their country of origin. Ongoing connection with a community from their country of origin provided the WoRB with connection to their culture and language of origin

‘It makes me so happy like for example, the first time we were in hospital, when I hear <language of country of origin>, I was like what? you are from <country of origin>, I was so happy, here are my people, I have people, because we do not have enough English to say the truths… but when people can understand your language it can make you happy’ – WoRB 2

‘We were kinda an early family, it got easier once more people started to be resettled from the background that we come from, and so then we could share each other’s issues with each other and get help if needed’ – WoRB 6

‘Now we have much more people who are from our background and who can speak our language, so we often gather up and do lots of activities, like social events and different festivals and getting together. This helps us feel really connected with each other’ – WoRB 9

Despite identifying that having a connection with individuals from the same community of origin is important for wellbeing and coping during resettlement, WoRB expressed that this is often difficult due to a lack of critical mass, particularly if individuals of refugee background leave regional areas due to lack of support and limited job prospects

‘When we came, we came with just 4 families, without any other people from [identified country of origin] but two families have gone, and one family last year have gone, and we are still here’ – WoRB 5

‘I think particularly in Tasmania, like community – small community groups is really hard. We see a lot of people transferring interstate recently actually. There has been a big pull to the big city, more work, bigger community means, um, bigger support networks. So the support networks in Hobart are limited’ – Service Provider 4

‘And the lack of numbers sometimes at the end of the day and a lot of people are moving interstate because there’s a lot more flattering options for employment and housing, whatever the draw card might be. They might have been here for 2months, maybe here for 20years and they are packing up because the mainland’s offering better options – Service Provider 12.

The lack of critical mass meant that many WoRB found a new community and support *via* connecting with their host community, with some WoRB choosing to engage with the host community for support due to experiencing ostracism from their own community of origin during resettlement.

‘I used to think that it was important that people got together with people from their community they could support each other. And it did not take long for me to realise that many individuals actually do not want to be part of their ethnic community here in Australia for lots of reasons. But, at the time, you know, I remember being a bit naïve about that’ – Service Provider 2

‘I made the assumption that she had adequate social supports because there we had a nice sort of, a number of other families from [her country of origin], but then down the track I realised that she was actually vilified or ostracised a bit by the other women because she’d been a widow, and alone with children, so I sort of asked her, I said, “Do you want me to find supports from Australian women?” and she was almost like, she looked at me like, “Ah, finally, yes that is what I want”’ – Service Provider 12

This connection with the host community was identified as initially stemming from the support of a volunteer, who not only provided practical support during the initial resettlement stages but often were a key source of connection and social support

‘The volunteers were very helpful. When we first came to Australia, we do not have a car, and we did not know where to go, so they came and picked us up, or helped us, and helped deliver people to hospital, school, anywhere’ – WoRB 5

‘Volunteers is such a huge part of like someone’s settlement journey, so having that kind of – I guess, volunteer or host family or a person in the community who is not of your culture, necessarily, to kind of walk you through and be with you’ – Service Provider 3

‘They become–they become friends really, you know’ – WoRB 2.

#### Sense of Meaning and Contribution

WoRB emphasised that contributing to the community in their resettlement location provided them with a sense of meaning and belonging, which in turn improved their wellbeing and coping during resettlement.

‘In my opinion, you only really start to feel settled when you get into a regular rhythm every day, so for me, I feel like I’m only starting to feel settled now, because I have work and I’m making some income, so that makes me feel a lot better about things, and feeling a little more normal’ – WoRB 1

The WoRB in the current study highlighted that this sense of belonging and contribution stemmed from gaining employment, which not only provided them with additional income, but connection and social support from the wider community.

‘Getting a job, and not for money, like for killing time, so your not sitting idle, so you can get up in the morning and get dressed and say, ‘oh I have a job’, it is very important for your mind, and it is good…. Like for me, it is important that I am not in the house for 24h – WoRB 2

‘A job – for the money, but also connection, so I am able to talk with people and feel useful. So I am able to meet people and I am not lonely and alone during the day’ – WoRB 4

‘A job is really important, because of income, but also it gives us a sense of meaning and something to do’ – WoRB 7

Overall, WoRB resettled in regional Australia identified several key factors which contributed to their coping and wellbeing during resettlement, including engaging in religious practices, finding a sense of community and having a sense of meaning and way to contribute within the community. Despite this, it was also identified that WoRB often struggled to access and engage in these factors, due to significant government oversights, lack of critical mass and lack of ongoing job opportunities.

## Discussion

This study explored the concept of resilience, and factors that contributed to wellbeing and coping in WoRB resettled in regional Australia. Twenty one interviews were conducted with WoRB and service providers supporting WoRB, with thematic analysis used to analyses the data. Results revealed insight into not only how WoRB understand resilience, but also factors that are critical to coping and wellbeing during resettlement.

### Exploring the Concept of Resilience in WoRB

The results from the first section of the study highlighted the difficulty of studying the concept of ‘resilience’ in WoRB, as the majority of the WoRB whom participated expressed their lack of understanding what ‘resilience’ was. This finding builds on research conducted by Pearce et al. (2017) which highlighted that traditional conceptualisations of resilience and associated theoretical models may have limited applicability to minority, non-Western populations, such as WoRB (Pearce et al., 2017; Hawkes et al., 2020). More specifically, this research goes beyond Pearce et al. (2017) research and uses WoRB’s historical and contemporary experiences to identify factors that should be included in a framework for developing a more appropriate conceptualisation of resilience for WoRB, which is discussed further below.

The WoRB who participated in the current study who showed an understanding of ‘resilience’ identified it as a concept associated with coping and moving forward. This challenges the traditional conceptualisation of resilience, which includes elements of ‘bouncing back’, absorbing or recovering (Panter-Brick et al., 2014; Hawkes et al., 2020), and aligns more closely with more contemporary conceptualisations, which acknowledges the dynamic nature of resilience, which involves a process of moving through adversity (Denckla et al., 2020) Within the current research, moving forward was associated with ‘everyday tasks’ rather than large life events, with WoRB indicating that their past experiences were a source of motivation and assisted them to move forward within the resettlement process. This is consistent with research carried out by Lenette (2011) which identified the ‘everyday-ness’ of resilience in WoRB, as they experienced adversity and challenges in completing everyday tasks. Within this, adversity was simply a part of their daily realities. Building on Lenette (2011) research, this research uniquely highlights how these adversities in everyday life are amplified when being resettled to a regional location of Australia. Regional resettlement contexts challenge WoRB across multiple facets of wellbeing, including their capacity to access practical necessities (employment, basic living needs and housing), but also spiritual (places of worship) and social (lack of social and cultural connection opportunities) needs. The daily challenges and adversity that WoRB resettled in regional Australia face deserves more acknowledgement, as to does the commitment shown by WoRB to moving forward and dealing with constant challenges across a prolonged period of time.

Overall, future research should be mindful of the non-critical application of ‘resilience’ in research focusing on refugee populations, and minority, non-Western populations in general (Ungar, 2005; Lenette et al., 2013). Developing and implementing a culturally grounded resilience framework, which can account for the aforementioned factors not only identified in the current study, but previous research, offers the potential of a more appropriate approach to anticipating the needs of WoRB and facilitating their adaptation. Developing a culturally grounded framework also represents the opportunity to inform future research questions in an area that is becoming increasingly important in countries taking in WoRB. Developing such a framework should be a focus of future research.

### Factors Contributing to Wellbeing and Coping During Resettlement

The second section of this study investigated factors that enhance coping and wellbeing in WoRB resettled in regional Australia. WoRB emphasised how a connection to their faith and ability to practice their religion was a core factor in their wellbeing and coping during resettlement. This is consistent with a growing body of research which has highlighted that religion and faith are the most commonly endorsed factor contributing to resilience and coping in WoRB during resettlement (see Hawkes et al. (2020), for a review). WoRB and service providers in the current study highlighted and identified a lack of access to places of religious worship [or no place of worship at all], in the regional locations of Australia that WoRB were resettled. This lack of consideration of factors of which increase the likelihood of positive resettlement outcomes can be identified as a gross oversight from a policy and practice perspective, as attending places of worship has not only been identified as a positive coping mechanism factor in previous research, but has also been identified as having positive flow-on effects, as it assists WoRB to build resources and social support networks (Gakuba, 2015).

‘Finding a community’ was identified by WoRB as playing an integral role in their coping during resettlement in regional Australia. For many WoRB, it was identified that connecting with a community from their country of origin provided them with an ongoing connection with their culture and language of origin. This is consistent with previous research by Sesay (2015) and Chung et al. (2013), which identified that ongoing connection with a community from their culture of origin provided WoRB with an identify and self-worth while navigating a new environment and informal support systems. Despite this, the current study identified that this is particularly difficult in regional locations due to a lack of critical mass. Critical mass is the term given when a number of individuals from a similar ethnicity are resettled in the same regional area. Critical mass has been identified as being imperative in regional resettlement locations, to not only enhance the provision of services in the area, but to allow in-kind social support, comfort and a sense of belonging (Shepley, 2007). This finding in conjunction with previous research (Shepley, 2007; Joyce and Liamputtong, 2017) highlights the importance of considering critical mass in a meaningful way (not just the arbitrary allocation of several WoRB to the same regional location) when planning and implementing regional resettlement from not only a service provision perspective, but also a wellbeing perspective.

Service providers highlighted that WoRB also found a ‘community’ by developing relationships with the host community. Developing social supports and ‘community’ with the host community were identified as the preference for some WoRB due to being ostracised by their community of origin during resettlement. WoRB resettled without male partners have been identified as being at particular risk of being ostracised by members of their community of origin due to social tensions stemming from cultural values (Schweitzer et al., 2018). For these WoRB, the establishment of ongoing connections with the host community was typically through volunteers. Volunteers have been identified as playing a significant role in establishing connections with the community and reducing isolation and disconnection during resettlement (Flanagan, 2007; Sypek et al., 2008). Volunteers fill a gap in support following resettlement due to government and non-government services in rural and regional areas of Australia being under-resourced (Sawtell et al., 2010). Gaining a deeper understanding into the support needs of volunteers supporting refugee populations during resettlement warrants further research, as to date, a majority of research focusing on service provision and supports during resettlement have not considered volunteers voices and perceptions, despite being at the coal face of interaction with newly arrived refugee populations (Sawtell et al., 2010).

WoRB also identified that a ‘sense of belonging and contribution’ enhanced their wellbeing during resettlement, gained particularly through employment. The benefits of gaining employment are multifaceted, and included the opportunity to earn extra income, but also provide WoRB a sense of purpose, routine and social support. Despite this, securing employment in regional locations has been identified as a significant barrier for refugee populations, despite this being a core objective of the regional resettlement scheme by the Australian government (to address employment shortages; Shergold et al., 2019). Satisfactory employment opportunities have been identified as critical indicators for successful resettlement in regional contexts and has been identified as one of the most significant challenges in regional resettlement contexts (Curry et al., 2018). The ability for WoRB to find employment in regional resettlement locations should be of particular concern to policy makers, as it is not only an indicator of long-term wellbeing, but also long-term retention of refugee populations to regional contexts. From a policy and programs perspective, investment into training and mentorship programs to increase employment opportunities and reduce the barriers to individuals of refugee background gaining employment in regional locations would help redress the situation (Curry et al., 2018). In addition to this, from a gendered perspective, policy and program planning will need to take into consideration the additional support needs that WoRB may require to gain employment, taking into consideration their additional education needs and childcare responsibilities (Smith et al., 2020).

### Limitations

The current study has several limitations. Firstly, a section of this research aimed to research the concept and understanding of the term ‘resilience’ in WoRB. For many of the women who participated in this research, they were unable to define the term. Although this is a valuable finding within itself, it may have also been hindered by the research being conducted in English. Secondly, the current study did not collect participant demographic information. This was a deliberate decision by the research team to reduce the likelihood of participants being identified, and breaching confidentiality. This is particularly pertinent in the current study due to it being conducted in a regional location, with some ethnic groups only having two-three families living in the regional location. Thus, identifying that a WoRB from that ethnic group participated in the study would have significantly reduced anonymity. Despite this, collecting and reporting demographic information would have provided important information pertaining to the generalizability of the current study population to future research; thus, it must be acknowledged as a limitation. Finally, the current research focused on WoRB resettled in a regional location in Australia; thus, the findings may have limited applicability to locations outside regional Australia. Despite this, the findings may provide insight into factors that need to be taken into consideration when resettling refugee populations to regional locations internationally, as similar challenges, including lack of critical mass and difficulty in finding employment, may impact on their long-term wellbeing.

### Conclusion

The findings of the current study support the need for research to move away from the application of Western-centric resilience frameworks in research focusing on non-Western populations and advance the understanding of adaptive experiences of WoRB during resettlement. The current study not only identifies factors that are identified by WoRB as being core to their coping and wellbeing during resettlement but also highlights the lack of consideration of these factors from a policy perspective. By identifying how many WoRB would be unable to access the identified coping factors where they were resettled, the continued use of Western-centric resilience frameworks may, at worst, hinder WoRB coping, adaptation and the quality of their settlement and development experiences. It is thus important that future research and practice develop theories and intervention strategies developed from WoRB accounts of their socio-cultural beliefs and practices rather than trying to fit them into a one-size-fits-all western model. The anticipated growth in the numbers of WoRB over the coming years and decades makes this a research and professional imperative.

## Data Availability Statement

The datasets presented in this article are not readily available due to ethical restrictions related to protecting the privacy imposed by the University of Tasmania and Charles Darwin University Research Ethic Boards, and the full, qualitative dataset (transcripts and field notes) cannot be made public. Public availability would compromise participant confidentiality and privacy. Requests to access the datasets should be directed to clare.hawkes@students.cdu.edu.au.

## Ethics Statement

The study was conducted according to the guidelines of the Declaration of Helsinki by the Institutional Review Board (or Ethics Committee) of Charles Darwin University (protocol code H19003 and H19087, approved January 2019 and March 2020, respectively) and University of Tasmania (protocol code H0017941 and H0020021, approved March 2019 and May 2020, respectively). The patients/participants provided their written informed consent to participate in this study. Informed consent was obtained from all subjects involved in the study.

## Author Contributions

CH, KN, JJ, and DP: conceptualization, methodology and writing – review and editing. CH: formal analysis, writing – original draft preparation and project administration. KN, JJ, and DP: supervision. All authors have read and agreed to the published version of the manuscript.

## Conflict of Interest

The authors declare that the research was conducted in the absence of any commercial or financial relationships that could be construed as a potential conflict of interest.

## Publisher’s Note

All claims expressed in this article are solely those of the authors and do not necessarily represent those of their affiliated organizations, or those of the publisher, the editors and the reviewers. Any product that may be evaluated in this article, or claim that may be made by its manufacturer, is not guaranteed or endorsed by the publisher.
